# Multi-Target Vehicle Tracking Algorithm Based on Improved DeepSORT

**DOI:** 10.3390/s24217014

**Published:** 2024-10-31

**Authors:** Dudu Guo, Zhuzhou Li, Hongbo Shuai, Fei Zhou

**Affiliations:** 1 The School of Transportation Engineering, Xinjiang University, Urumqi 830017, China; guodd@xju.edu.cn; 2 Xinjiang Key Laboratory of Green Construction and Smart Traffic Control of Transportation Infrastructure, Xinjiang University, Urumqi 830017, China; 3 The School of Intelligent Manufacturing for Modern Industry, Xinjiang University, Urumqi 830017, China; shuaihongbo@126.com (H.S.); 107552201426@stu.xju.edu.cn (F.Z.)

**Keywords:** optical satellite images, vehicle detection, YOLOX, scale expansion, attention mechanism, BFPnet

## Abstract

In this paper, we address the issues of insufficient accuracy and frequent identity switching in the multi-target tracking algorithm DeepSORT by proposing two improvement strategies. First, we optimize the appearance feature extraction process by training a lightweight appearance extraction network (OSNet) on a vehicle re-identification dataset. This makes the appearance features better suited for the vehicle tracking model required in our paper. Second, we improve the metric of motion features by using the original IOU distance metric or GIOU metrics. The optimized tracking algorithm using GIOU achieves effective improvements in tracking precision and accuracy. The experimental results show that the improved vehicle tracking models MOTA and IDF1 are enhanced by 4.6% and 5.9%, respectively. This allows for the stable tracking of vehicles and reduces the occurrence of identity switching phenomenon to a certain extent.

## 1. Introduction

Vehicle detection and tracking is a crucial technology for autonomous driving and intelligent transportation systems. It aims to enable intelligent systems to analyze and understand vehicle behavior in video sequences, gather semantic information, and achieve vehicle detection and tracking without human intervention or with minimal human intervention [1]. The application of this technology can enhance the safety of vehicles during autonomous driving, manage intelligent transportation systems on a daily basis, and respond promptly to emergencies. This, in turn, improves road traffic safety and mobility and optimizes the driving environment [2,3].

The tracking of multiple objects (MOT) is a fundamental task in computer vision that finds wide applications in areas such as autonomous driving, surveillance, and behavior recognition [4]. The accurate detection and tracking of multiple targets are essential for various fields and applications [5,6]. However, tracking multiple targets presents significant challenges, particularly in scenarios involving complex traffic flows where issues like occlusion, motion blur, and high appearance similarity make robust tracking more difficult. In the realm of intelligent transportation, multi-vehicle tracking is a crucial component of the intelligent transportation system.

Vehicle tracking is essential for efficient and stable speed detection in intelligent transportation [7]. Traditional target-tracking methods rely mainly on computer vision and image processing technology. These methods prioritize manual feature extraction and target model creation over deep learning-based approaches. Examples of such methods include the Mean Shift Filter, Kernelized Correlation Filter, and other correlation filter-based techniques; the corner detection algorithm; scale-invariant feature transform (SIFT); the accelerated robust feature (SURF); as well as motion model-based methods such as Kalman filtering [8]. The traditional method of target tracking works well in simple scenes but faces challenges with target appearance changes, occlusion, and complex dynamic scenes [9]. With the advancement of deep learning technology, object-tracking methods based on deep learning have become the norm due to their high accuracy and robustness. To address the limitations of traditional appearance-learning methods in distinguishing multiple targets with significant appearance changes, Bae et al. [10] proposed a deep appearance-learning strategy. This method uses large training datasets to build a discriminative appearance model, thereby enhancing the reliable connection between trajectory and detection. Additionally, the method integrates online transfer learning technology to enhance appearance discrimination by making real-time adjustments to the pre-trained deep model during the tracking process. Kim et al. [11] introduced an efficient online visual Multiple Object Tracking (MOT) algorithm that combines detection-based and TBD (to be determined) methods. This algorithm integrates state estimation, trajectory management, clutter suppression, false negatives, and occlusion processing into a Bayesian recursion, enhancing the real-time performance of multi-target tracking algorithms. Yang et al. [12] proposed a method that applies compressed sensing features to the Markov Decision Process (MDP) tracking framework. The method includes the design of a single target tracker, the combination of compression tracking and optical flow tracking, and the use of a Kalman filter for prediction to improve the speed of local sampling when searching for the target location and size. This tracker was then integrated into the MDP multi-target tracking framework, enhancing feature representation in data association by updating the discriminant appearance model using P-N learning for each target. Given the challenges posed by environmental complexity and changes in lighting and scale, Qiu et al. [13] proposed a moving vehicle tracking algorithm based on deep learning. This algorithm includes an improved traditional GMM algorithm to reduce the misjudgment probability of the pixel state. Additionally, a sparse DAE (Denoising Autoencoder) neural network feature learning framework is proposed to efficiently extract vehicle features, reduce feature redundancy, and improve the robustness of the tracking algorithm. To reduce the burden of manual labeling and realize the tracking learning of arbitrary objects, Wang et al. [14] proposed a visual tracking method based on unsupervised learning. This method uses a Siamese correlation filter network to construct a tracker. It introduces a multi-frame verification scheme and cost-sensitive loss function to promote the progress of unsupervised learning. This approach allows unsupervised trackers to achieve baseline accuracy comparable to traditional fully supervised trackers while maintaining real-time speed. Lee et al. [15] proposed a monocular MOT system that achieved high-precision and high-efficiency tracking tasks by utilizing the improved EfficientNet backbone network and an efficient FPN-based output feature fusion. The experimental results indicate that the developed multi-target tracking system outperforms most state-of-the-art trackers on the UA-DETRAC dataset and operates at high inference speeds. Peng et al. [16] proposed a constrained neural architecture search (Constrained-NAS) method for multimodal fusion, aimed at completing multi-object tracking tasks within specified time constraints. In the first phase, an efficient feature extraction backbone is searched, while the second phase involves model training based on the identified architecture. The effectiveness of this approach was validated on the KITTI benchmark test, achieving an accuracy of 89.59%, which is close to state-of-the-art methods, while maintaining latency below 80 ms. This demonstrates both high accuracy and real-time performance.

In the realm of intelligent transportation, multi-target tracking technology has garnered significant attention and research, particularly in vehicle tracking. It has been proven to effectively enhance the efficiency and safety of traffic management. While traditional target tracking methods are effective in simple scenarios, multi-vehicle tracking stands as a crucial component of an intelligent transportation system [17]. In dense traffic flows, challenges such as occlusion, motion blur, and high appearance similarity make reliable target tracking more difficult. Conversely, multi-target tracking based on deep learning offers numerous advantages. Deep learning methods can automatically learn and extract features without the need for manual design and feature selection, thereby simplifying the model construction process. Moreover, these methods can handle large-scale data and provide more accurate and stable tracking results for complex traffic scenarios with numerous vehicle targets [18]. Additionally, deep learning methods exhibit strong generalization ability, making them adaptable to various traffic environments and conditions. In summary, the integration of deep learning-based multi-target tracking methods can offer more efficient and accurate solutions in the field of intelligent transportation, especially in vehicle tracking, ultimately enhancing traffic management efficiency and road safety [19].

This paper begins by introducing the DeepSORT multi-target tracking algorithm and examining the challenges of vehicle tracking in complex traffic environments. To address issues such as the low tracking accuracy and frequent ID switching of multi-target vehicles in dense scenes, the DeepSORT algorithm was enhanced. This involved using the OSNet as the base network to improve appearance extraction features and retraining the vehicle re-identification network by migrating the target feature extraction network originally designed for pedestrian recognition. Additionally, to enhance the matching capability of the tracking model, improvements were made to the IOU distance metric. The model was then tested using the converted UA-DETRAC tracking dataset, and the resulting experimental data were comprehensively analyzed to evaluate the model’s ability to track multiple vehicles.

## 2. Vehicle Detection Algorithm for Complex Traffic Scenarios

In complex traffic scenes, vehicle detection faces many challenges that mainly stem from the diversity of the environment, the complexity of the scene, and the variability of the vehicles themselves. Some of the main challenges include the following: (1) The occlusion problem: In crowded traffic environments, vehicles often obscure each other, partially or completely hiding other vehicles. This occlusion phenomenon brings great difficulty to vehicle detection and tracking. (2) Scale change: Distant vehicles will appear smaller and contain less information than nearby vehicles, even if they are of the same model and size. This scale change requires the detection algorithm to accurately identify vehicles at different scales. (3) Real-time requirements: For applications such as autonomous driving systems, vehicle detection must not only be accurate but also fast enough to handle rapidly changing road conditions in real time. Increasing the number of network layers can improve the detection speed, but it can also greatly reduce the detection speed. Meeting real-time demand while ensuring accuracy is also an urgent problem to be solved. (4) Dynamic background: In high-speed moving scenes, the background is also changing rapidly, which may interfere with vehicle detection. Additionally, weather conditions such as rain, snow, and fog can change the visual characteristics of the scene, increasing the difficulty of detection.

**Figure 1:**
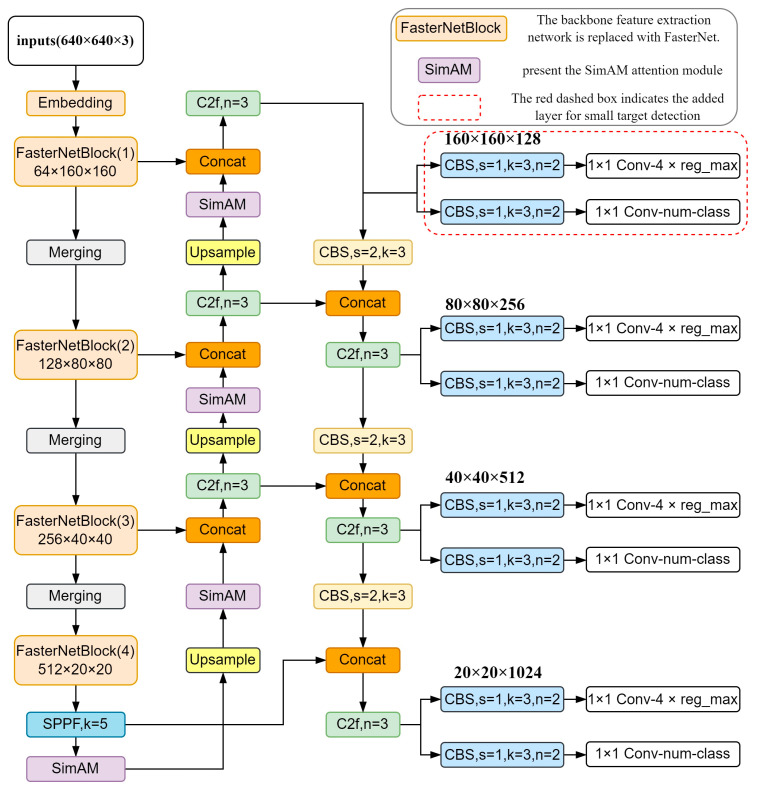
Improved YOLOv8 network structure.

In challenging traffic scenarios, detecting vehicles can be difficult, leading to problems like false detections and a lack of real-time performance. To address this, our paper proposes improvements to the YOLOv8 target detection algorithm as shown in Figure 1. We enhanced the network structure by performing the following:

(1) Replacing part of the backbone network with the lightweight FasterNet to boost the model detection speed;

(2) Introducing the non-parametric attention mechanism SimAM to enhance important vehicle features;

(3) Adding a small target detection head of 160 × 160 to improve accuracy in detecting small vehicles in complex traffic scenarios;

(4) Introducing WIOUv1 to optimize the training process and improve the model’s predictive regression performance on boundary boxes.

### 2.1. Optimizing the Feature Extraction Network

To accommodate different detection needs, YOLOv8 models, based on YOLOv5, offer strategies for multiple scales with five model variants of different sizes: n/s/m/l/x. To prevent the unnecessary use of computing resources due to overly complex models for simple tasks, and to maintain the detection speed, this research takes into account both detection accuracy and speed. It starts with a relatively lightweight small model as a baseline for improvement.

**Figure 2:**
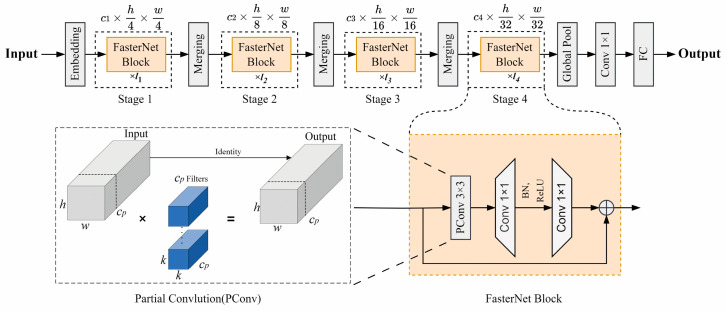
The network structure of FasterNet.

To further enhance performance, this research replaces the original CSPDarknet backbone feature extraction network with the FasterNet network. FasterNet’s key feature is the use of partial convolution (PConv) operations, which reduce redundant computations and memory accesses to efficiently extract spatial features. This enables higher running speeds on a variety of devices while maintaining accuracy for different visual tasks. The network structure of FasterNet is shown in Figure 2 [20].

### 2.2. Add a Small Target Detection Header

The original YOLOv8 model uses a relatively large image downsampling ratio to provide a wider receptive field. This allows the model to perform well in predicting large-scale targets. However, this approach results in sparse features for small targets in the receptive field, limiting the model’s ability to accurately detect and locate them. This limitation becomes particularly noticeable when dealing with small targets, as important details can be lost during downsampling.

Given that the public dataset used contains many complex traffic scenes with densely distributed small and heavy blocking vehicles, this paper proposes adding a small target detection layer based on the original head layer. This new layer, based on a 160 × 160 feature map, is designed to capture more detailed information about the original image, enabling the effective identification of target vehicles away from surveillance cameras. The specific structure is shown in Figure 1.

In this paper, the 80 × 80 feature map of the second layer of FasterNetBlock in the backbone network is combined with the feature layer of the Neck layer after upsampling. Through the C2f module and the upsampling step, a layer rich in small target feature information is formed. This layer is further combined with the 160 × 160 feature map output by FasterNetBlock, the first layer of the backbone network, in the channel dimension. This greatly strengthens the ability of the fusion feature map to capture small targets and the sensitivity of the network. Additionally, the C2f module outputs a detection head specifically tailored for small targets, further enhancing the positioning accuracy of the model.

### 2.3. Introducing the SimAM Attention Mechanism

**Figure 3:**
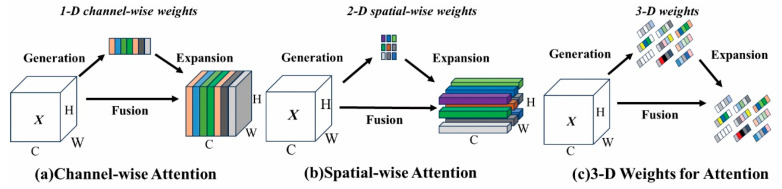
SimAM structure.

This paper introduces the Simple, parameters-free Attention Module (SimAM) mechanism to capture key information about a target vehicle in a complex traffic scene and improve the representation ability of the model. Unlike traditional attention mechanisms, SimAM can calculate attention weights in three dimensions for feature graphs, allowing for the interpretability of the model’s working mechanism. Importantly, SimAM is parameter-free, which is crucial for designing lightweight networks. The attention module’s structure is depicted in Figure 3 [21].

The main idea behind SimAM is to selectively enhance or suppress specific areas of a feature map to emphasize the features that are more important for downstream tasks. SimAM achieves attention modulation through the following steps:

(1) Feature transformation: Calculate the importance score of each position in the feature map using a simple transformation form.

(2) Feature scaling: Transform the original feature map using the importance score obtained from the previous step through a simple scaling function, which might involve a nonlinear transformation based on the score of the feature and a preset threshold.

(3) Feature reweighting: Use the importance score obtained after scaling to weigh the original feature map, which will strengthen the response of some feature points, enabling the network to focus on more important features.

### 2.4. Optimizing the Loss Function

In object detection algorithms, the loss function is a crucial element that directly impacts the model’s learning process. A well-designed loss function not only speeds up model convergence but also enhances its ability to generalize and improve accuracy in complex scenarios. The original model used the CIoU [22] loss function for boundary frame regression, effectively considering factors like the aspect ratio and distance between the predicted and real frames, leading to significant improvements in positioning accuracy. However, this loss function has limitations when dealing with small or low-quality label frames.

To address these limitations, the EIOU (Focal Efficient Intersection over Union) [23] loss function was introduced, incorporating a static focusing mechanism to enhance problem instances. Despite this improvement, the potential of its non-monotonic behavior was not fully utilized. Subsequently, the *WIoU* (Wise Intersection over Union) [24] loss function was proposed. This loss function evaluates the anchor frame quality through a dynamic non-monotonic focusing mechanism, transitioning from *IoU* evaluation to outlier evaluation. Its goal is to reduce the excessive punishment of geometric factors and minimize training process interference to enhance model generalization. In summary, the unique design of the *WIoU* loss function balances geometric parameter optimization and model generalization, strengthening the model’s regression performance for small targets and low-quality label boxes.

(1) $$L_{IoU}=1-IoU=1-\frac{W_{i}H_{i}}{S_{u}}$$

(2) $$L_{WIoUv1}=R_{WIoU}L_{IoU}$$

(3) $$R_{WIoU}=\exp(\frac{{\left(x-x_{gt}\right)}^{2}+{\left(y-y_{gt}\right)}^{2}}{{\left(W_{g}^{2}+H_{g}^{2}\right)}^{*}})$$

**Figure 4:**
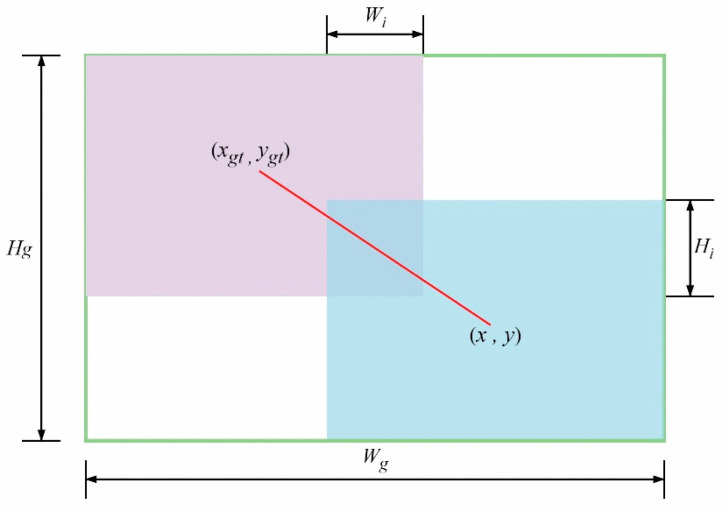
Schematic diagram of prediction box and real box.

In the equation, *W_g_* and *H_g_* represent the width and height of the minimum external frame of the prediction box and the real box, respectively. *W_i_* and *H_i_* represent the width and height of the intersection area of the prediction box and the real box, while *Su* represents the joint area of the prediction box and the real box. A schematic diagram of the prediction box and the real box is shown in Figure 4. To prevent the creation of gradients that impede convergence, *W_g_* and *H_g_* are separated from the computed graph, denoted by the superscript *. This operation effectively eliminates factors that hinder convergence, avoids the introduction of new metrics such as the aspect ratio, and reduces the negative impact on the convergence of the aspect ratio function in the original CIoU loss function.

The Wise-*IoU* loss function considers both the relative position and size difference between detection targets and includes an intelligent weight adjustment mechanism. This mechanism enhances the flexibility and robustness of the vehicle detection task by automatically adjusting the weight coefficient. To further improve the detection performance of the enhanced model, this paper chooses *WIoU*v1 as a loss function for model optimization. Later on, *WIoU*v1 will be referred to simply as *WIoU*.

## 3. DeepSORT Multi-Target Tracking Algorithm

**Figure 5:**
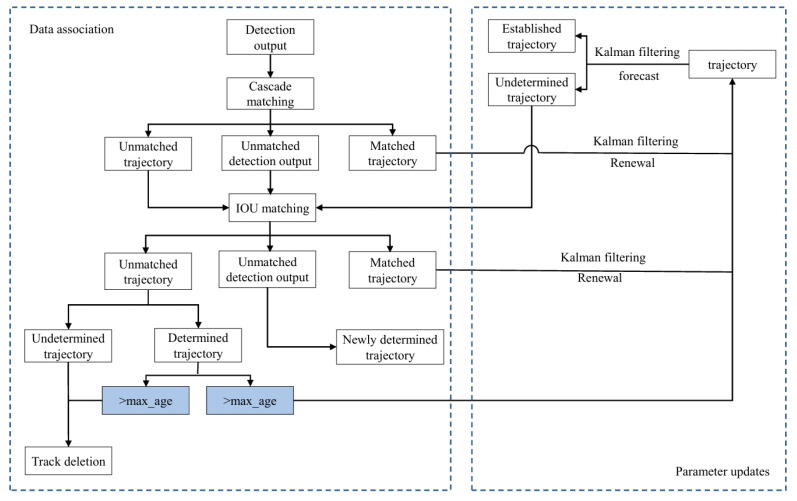
DeepSORT algorithm flow.

The DeepSORT (Deep Simple Online and Realtime Tracking) algorithm [25] is widely used in the field of multi-target tracking. It is an extension of the SORT (Simple Online and Realtime Tracking) [26] algorithm designed to enhance tracking performance by incorporating deep learning. DeepSORT effectively combines appearance and motion information to achieve real-time multi-target tracking. Specifically, it integrates a deep learning model into the SORT algorithm to extract target features, thereby improving the accuracy of target recognition and matching during the tracking process. The flow chart of the DeepSORT multi-target tracking algorithm is displayed in Figure 5.

In Figure 5, the DeepSORT algorithm builds on the SORT algorithm by adding cascade matching and new trajectory confirmation. The overall process is as follows:

(1) The Kalman filter is used to predict the state of each target.

(2) The Hungarian algorithm is used for global optimal matching. It constructs a cost matrix containing similarity scores between the targets and finds the matching scheme with the lowest cost, representing the best matching between the targets.

(3) Each target’s state in the Kalman filter is updated according to the matching result.

### 3.1. The Estimation of the Target State

The Kalman filter algorithm is a key component of the multi-target tracking algorithm DeepSORT, which is primarily used for estimating and predicting the motion state of a target vehicle. This algorithm combines prior information (such as the target’s previous state) with measurement information (such as the target’s detection result) to estimate and predict the target’s state. It provides the current position and speed information of the target and also predicts the target’s future motion state, enabling effective tracking and prediction. The algorithm defines the target’s motion state using eight normal distribution vectors.

(4) $$\left(u,v,r,h,\overset{\cdot }{x},\overset{\cdot }{y},\overset{\cdot }{r},\overset{\cdot }{h}\right)$$

(*u*,*v*) represent the coordinates of the center point of the target vehicle detection frame. “*r*” represents the aspect ratio of the detection frame, “*h*” represents the height of the target detection frame, and the quadruple (*x*,*y*,*r*,*h*) describes the relative speed information in the image coordinates. In the process of moving the target, the position and speed parameters of the target frame in the current frame can be predicted based on the parameters of the target frame and its speed in the previous frame.

The prediction process using a Kalman filter can estimate the location of the tracking frame at time “*t*” based on the tracking frame at time “*t* − 1”. The prediction process is described in Equations (2) and (3).

(5) $$X_{t}=FX_{t-1}$$

(6) $$P_{t}=FP_{t-1}F^{T}+Q$$

In the equation above, *X_t_* represents the prediction box at time *t*, *P_t_* represents the covariance matrix at time *t*, *Q* represents the noise matrix of the current filter, *F* is the state transition matrix as shown in Equation (4), and *dt* is the time frame difference between *t* and *t* − 1.

(7) $$F=\left[\begin{array}{cccccccc} 1 & 0 & 0 & 0 & dt & 0 & 0 & 0\\ 0 & 1 & 0 & 0 & 0 & dt & 0 & 0\\ 0 & 0 & 1 & 0 & 0 & 0 & dt & 0\\ 0 & 0 & 0 & 1 & 0 & 0 & 0 & dt\\ 0 & 0 & 0 & 0 & 1 & 0 & 0 & 0\\ 0 & 0 & 0 & 0 & 0 & 1 & 0 & 0\\ 0 & 0 & 0 & 0 & 0 & 0 & 1 & 0\\ 0 & 0 & 0 & 0 & 0 & 0 & 0 & 1 \end{array}\right]$$

Once the state prediction of the target vehicle is finished, the Kalman filter will then update the vehicle’s state. To carry this out, we need to calculate the Kalman filter gain *K_t_* of the target vehicle. The calculation expression for *K_t_* is shown in Equation (5).

(8) $$K_{t}=\frac{{\bar{P}}_{t}H^{T}}{H{\bar{P}}_{t}H^{T}+R}$$

At time “*t*”, *K_t_* represents the Kalman filter gain of the target vehicle, *R* represents the covariance of the measurement noise, and *H* represents the conversion matrix from the current motion state to the measured value. The covariance matrix is constant.

(9) $$H=\left[\begin{array}{cc} I_{4} & O \end{array}\right]$$

The most accurate estimate of the target state is determined using Equation (7).

(10) $$\hat{x_{t}}={\hat{x_{t}}}^{-}+K_{t}(z_{t}-H{\hat{x_{t}}}^{-})$$

The updated covariance matrix is displayed in Equation (8).

(11) $$P_{t}=(1-K_{t}H){\bar{P}}_{t}$$

The analysis indicates that the value of the Kalman gain is influenced by the covariance *Q* of the prediction estimate and the covariance *R* of the measurement noise. A larger *Q* means a noisier motion model and results in a larger Kalman gain, causing the updated target state to rely more on the observed *z_t_*. Conversely, a larger covariance matrix *R* for measurement noise leads to a greater observation error and a smaller Kalman gain value. This implies that the filter is less responsive to new measurements and relies more on the model’s predictions, resulting in the final updated target state being closer to the predicted value.

### 3.2. Data Association

In the DeepSORT algorithm, the data association step is crucial for ensuring that the same objects are connected across different frames. This step typically utilizes the Hungarian algorithm, a classical method for solving the Assignment Problem. When employing the Hungarian matching algorithm, the cost matrix is primarily determined based on two aspects. First, the cost matrix considers the spatial distance between the trajectory and the detection frame, calculated by introducing the vehicle motion model. The motion model predicts the vehicle’s position and compares it with the actual detected position to measure the spatial distance between the two. Secondly, the cost matrix contains the measure of the appearance model, which evaluates the appearance consistency between the trajectory and the detection frame. Comparing appearance features helps determine whether the detected objects in two different frames are the same target.

(1) Association of Movement Information

In the multi-target tracking algorithm DeepSORT, the Mahalanobis distance is utilized to assess the similarity between target features. This aids in associating the target detected in the current frame with the tracking target in the previous frame. The calculation equation is shown in (9). Unlike Euclidean distances, Mahalanobis distances consider correlations and covariances between data, making them more suitable for describing differences between data in high-dimensional spaces.

(12) $$d^{\left(1\right)}(i,j)={\left(d_{j}-y_{i}\right)}^{T}S^{-1}(d_{j}-y_{i})$$

where *d* represents the detection position of the *j* detected target, *y* represents the predicted position of the *i* tracker to the current target, and *S* represents the covariance matrix of the observation space at the current moment predicted by the Kalman filter. Based on the calculated Mahalanobis distance, a threshold can be set to determine whether the two target feature vectors are sufficiently similar. If the Mahalanobis distance is less than the threshold, the two objects are considered similar and can be associated. Otherwise, they are not considered similar and are not associated. The calculation equation is shown in (10).

(13) $$b_{i,j}^{\left(1\right)}=[d^{\left(1\right)}(i,j)]\leq t^{\left(1\right)}$$

The Mahalanobis distance is utilized in DeepSORT to quantify the similarity between target features. This helps in correlating a target detected in the current frame with a tracking target in the previous frame. Generally, the target feature is represented as a vector that includes the spatial position of the target, as well as appearance features.

(2) Association of Appearance Information

The motion information association typically only takes into account dynamic information, such as the position and speed of the target, while disregarding the appearance characteristic information of the target. This often leads to a high number of target identity switches or target loss. To address these issues, the DeepSORT algorithm incorporates appearance information association and utilizes the cosine distance to describe appearance features such as the color and texture of targets. This enhances the ability to distinguish between targets and reduces confusion and false associations. The calculation equation is presented in Equation (14).

(14) $$d^{\left(2\right)}(i,j)=\min\{1-r_{j}^{T}r_{k}^{\left(i\right)}|r_{k}^{\left(i\right)}\in Ri\}$$

The equation *d*^(2)^(*i, j*) is used to calculate the minimum cosine distance between the appearance feature vectors of the i-th tracking box and the j-th detection box. The variables *r_j_* and *r_k_*^(*i*)^ represent the feature vectors of the j-th detection target and the i-th tracker, respectively. The variable *r_j_^T^r_k_*^(*i*)^ calculates the cosine similarity, and 1 − *r_j_^T^r_k_*^(*i*)^ represents the cosine similarity between the two vectors.

The cosine distance is then used to determine whether the appearance information is successfully matched by comparing it to a specific threshold, denoted as *t*^(2)^. If the calculated cosine distance is lower than the threshold, the appearance information is considered to be successfully associated. The determination equation is shown in Equation (15).

(15) $$b_{i,j}^{\left(2\right)}=\prod \left[d^{\left(2\right)}(i,j)\leq t^{\left(2\right)}\right]$$

The data association step of the DeepSORT algorithm is crucial. It comprehensively considers both the motion and appearance characteristics of the target to make a final assessment. By combining the distance measurement from the motion model and the consistency score from the appearance model, we can calculate the overall matching score between the trajectory and the detected object using the equation shown in (16).

(16) $$\left\{\begin{array}{c} c(i,j)=\lambda d^{\left(1\right)}(i,j)+(1-\lambda )d^{\left(2\right)}(i,j)\\ b_{i,j}=\prod^{2}b_{i,j}^{\left(m\right)} \end{array}\right.$$

In the equation, parameters are utilized to adjust the balance between the Mahalanobis distance and cosine distance. A correct match is only achieved when *c*(*i*, *j*) satisfies both the criteria for appearance information and the criteria for motion information association simultaneously. When $b_{i,j}=1$, both appearance and motion information are involved in the calculation of similarity, and the preliminary match is considered successful.

### 3.3. Cascading Matching

**Figure 6:**
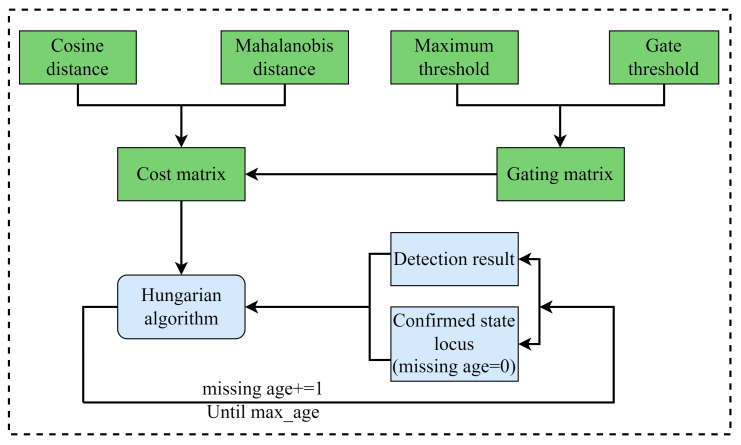
Cascade matching process.

Matching Cascade is a fundamental algorithm that sets Deep SORT apart from SORT. It aims to address situations where an object is obstructed or missing for an extended period, causing the detector to lose track of the object and the tracker to fail to match it with the previous trajectory. To ensure that the current detection matches the trajectory near the current time, the Deep SORT algorithm incorporates a cascade matching algorithm to link the target trajectory with more frequent occurrences in advance. The matching process is illustrated in Figure 6.

In the multi-target tracking algorithm, it is crucial to link the target detected in the current frame with the target tracked in the previous frame. As the number of targets increases, the computational workload for the association also increases, leading to reduced efficiency in the tracking algorithm. The DeepSORT algorithm addresses this challenge by simplifying the data association problem with a cascaded matching strategy, which effectively reduces the computational load and improves the tracking efficiency and robustness. This approach involves staging local matching and ADAPTS to the tracking requirements in complex traffic scenarios.

However, in cases where cascaded matching is inadequate, such as during long periods of occlusion or abrupt changes in the target’s appearance, DeepSORT introduces IOU matching to ensure continuity and accuracy in tracking.

## 4. Improved DeepSORT Multi-Target Vehicle Tracking Algorithm

### 4.1. Optimizing the Feature for Extracting Appearances

The DeepSORT multi-object tracking algorithm was originally designed for pedestrian tracking tasks, and its depth appearance descriptor module’s training weights were mainly optimized for pedestrian features. These weights were trained on the pedestrian re-recognition dataset Market 1501, so the model shows high sensitivity to pedestrian features. However, its performance in vehicle re-recognition tasks is relatively poor. Detailed information on the target feature extraction network structure of the original model is shown in Table 1.

The network structure information in Table 1 reveals that the original network structure is relatively shallow, resulting in certain limitations in the appearance feature information extracted from it. Since the training set is limited to pedestrian images and the size of the input feature extraction network is 128 × 64, this aspect ratio does not match the actual vehicle shape. In this paper, the OSNet (Omni-Scale Network), a lightweight full-scale network model, is chosen to extract vehicle appearance feature information. The network input size is optimized and adjusted to effectively meet the needs of vehicle feature extraction.

**Figure 7:**
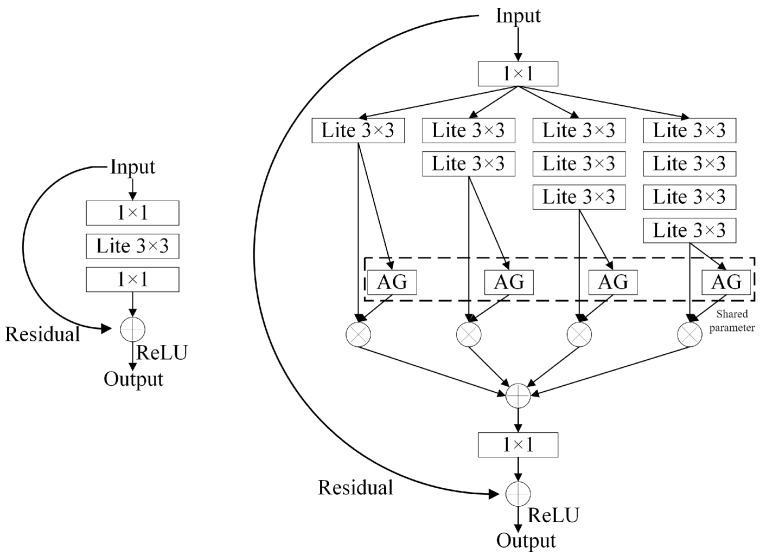
OSNet structure.

The introduction of the Omni-Scale module in OSNet allows the network to effectively handle scale changes in vehicle recognition tasks. This design enables the network to extract features at different scales, improving its adaptability. Additionally, OSNet uses a global context aggregation module to capture global semantic information, allowing it to effectively utilize the semantic information of the entire image. Furthermore, OSNet enhances feature extraction efficiency and generalization ability by optimizing network stride settings and applying label smoothing regularization. This results in a faster inference speed while maintaining high accuracy. Although the OSNet was initially designed for personnel recognition, its multi-scale feature extraction and global context aggregation mechanisms make it well suited for vehicle recognition tasks, demonstrating its strong versatility and applicability in the field. The OSNet structure is depicted in Figure 7 [27].

**Figure 8:**
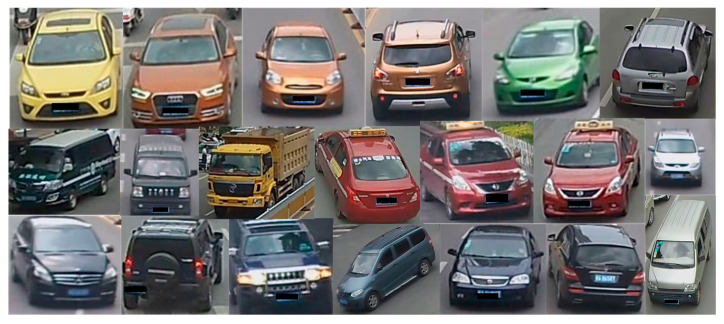
Sample training of VeRi-776 dataset.

To enhance the ability to extract specific vehicle appearance features, one approach is to fine-tune the appearance feature extraction network to accommodate different vehicle sizes and train it on a vehicle re-recognition dataset. This will enhance the performance of the multi-target vehicle tracking model. The VeRi-776 dataset contains over 50,000 photos of 776 vehicles captured by 20 cameras covering an area of 1 square kilometer within 24 h. An example of the dataset is shown in Figure 8. This rich dataset is highly scalable and suitable for vehicle re-recognition research and related fields. The images are captured in real-world, unrestricted surveillance scenarios and include diverse vehicle attributes such as Bboxes, vehicle types, colors, and brands. Such comprehensive datasets provide a solid foundation for developing and evaluating complex vehicle re-recognition models. Each vehicle in the dataset is captured by 2 to 18 different cameras, encompassing multiple viewpoints, various light conditions, resolutions, and occlusions, making the dataset highly realistic for simulating vehicle re-recognition challenges in a real-world monitoring environment.

The experimental environment setup is detailed in Table 2. The model’s hyperparameter settings are as follows: the input image size is 256 × 256, the Adam optimizer is used, the initial learning rate is 0.00035, the batch size for training is 40, and the number of training iterations is 126.

**Figure 9:**
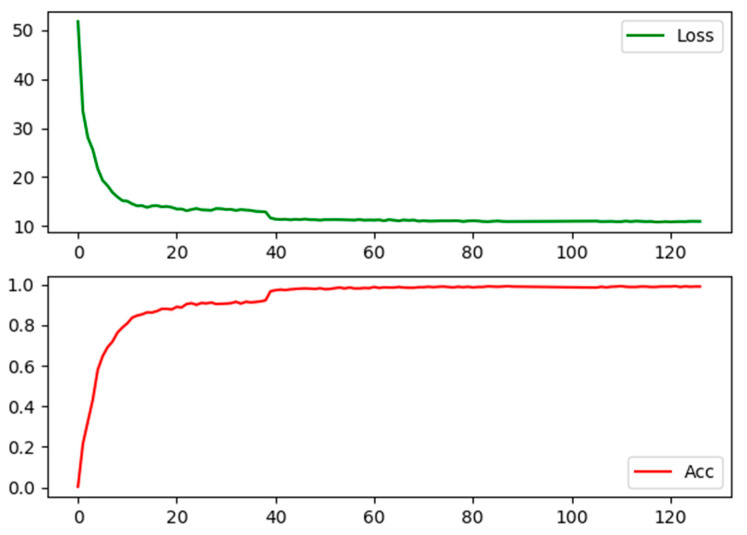
OSNet training process of vehicle re-recognition network.

The training parameters above are utilized for conducting appearance feature training on the VeRi dataset, with OSNet being used to extract vehicle appearance features. The training process is illustrated in Figure 9.

In Figure 9, we can observe the training process of the OSNet algorithm. During the initial 20 training cycles, the algorithm parameters are continuously updated, and the network layers of the OSNet quickly adapt to the vehicle recognition task. At this stage, the loss value shows a rapid decline. From the 20th to the 40th epoch, the rate of decline in the loss value gradually slows down, indicating that the model parameters can accurately identify the feature differences between different vehicles and the feature associations between the same vehicles. Similarly, in the training process reflected in Figure 9, we observe that in the first 40 training cycles, the model’s accuracy improves rapidly, approaching 0.90. Subsequently, with the steady progress of the training process, the accuracy rate gradually approaches 1, indicating that the algorithm’s performance has reached a stable state.

### 4.2. Enhancing the Measurement of the IOU Motion Feature

**Figure 10:**
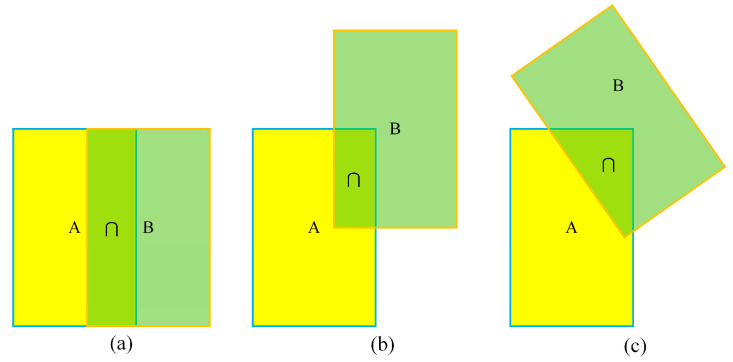
Example of prediction box overlapping with real box. (**a**–**c**) represent different intersections between the prediction box and the real box, respectively.

After the cascade matching process, while most tracks and detection boxes can be successfully matched, there are still some unmatched instances. To address this issue and ensure the correct association of these remaining tracks with the detection frame, a matching strategy based on the IOU distance is introduced. This strategy involves creating an IOU value cost matrix between the detection frame and the track frame and then using the Hungarian algorithm to find the best matching detection frame for each unassigned track, thus ensuring that it corresponds correctly to the actual tracking target. However, the IOU value has some limitations as a metric. For example, the same IOU value may correspond to multiple prediction boxes in different locations, which means that the IOU value cannot accurately reflect the intersection between the prediction box and the real box, particularly if the specific relative position of the prediction box cannot be determined. This aspect can affect the accuracy of vehicle tracking. Figure 10 demonstrates the potential limitations of the IOU metric when applied to vehicle tracking by presenting several cases where the prediction box overlaps with the real box.

**Figure 11:**
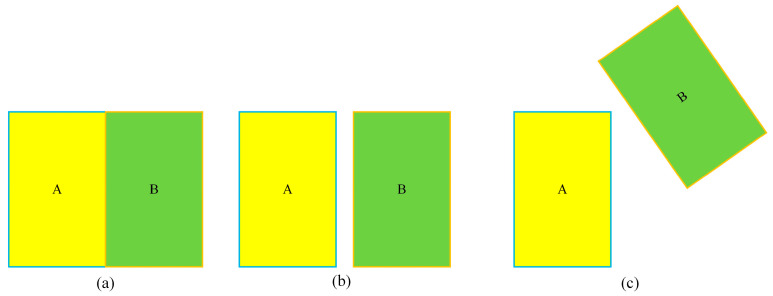
Example of prediction box and real box not overlapping. (**a**–**c**) indicates different degrees of adjacency.

**Figure 12:**
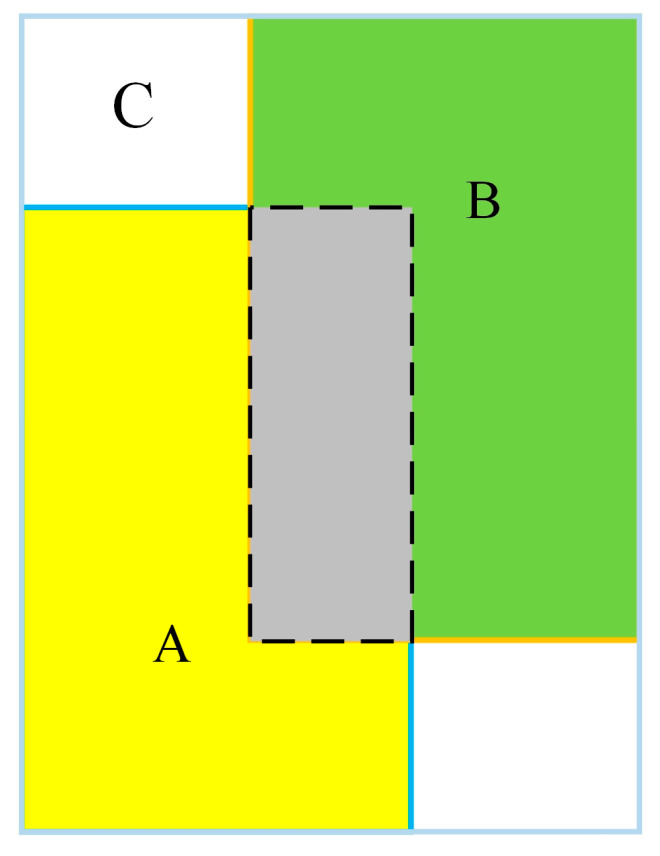
Example of GIOU metrics.

In Figure 11, when IOU = 0, it is not possible to accurately measure the degree of adjacency between the prediction box and the real box, which can lead to tracking inaccuracies. To address this issue, this paper proposes using the GIOU measurement method. By using GIOU as a new measurement indicator, this paper introduces the minimum external frame that includes the prediction frame and the real frame, effectively addressing the limitation of the traditional method in accurately reflecting the overlap degree of the two. The schematic diagram of the GIOU measurement is depicted in Figure 12, and its calculation method is illustrated in Equation (17).

(17) $$\left\{\begin{array}{c} \mathrm{IoU}=\frac{\left|A\cap B\right|}{\left|A\cup B\right|}\\ GIoU=IoU-\frac{\left|C\backslash (A\cup B)\right|}{\left|C\right|} \end{array}\right.$$

where C represents the smallest external rectangular box surrounding A and B, and C\(A∪B) represents the area of C minus the area of A∪B.

Due to the limitations of the IOU metric in accurately measuring the proximity and intersection between the detected vehicle and the tracked trajectory, this paper introduces GIOU as an alternative. GIOU not only reflects the proportion of intersecting parts but also takes into account the relative position information of the trajectory and detection box. This improves the matching ability of the DeepSORT tracking algorithm. The experimental results indicate that the tracking accuracy and effectiveness of the GIOU-enhanced tracking algorithm can be improved. Additionally, the probability of tracking failure can be significantly reduced when dealing with occlusion.

## 5. Analysis of the Experimental Process and Results

### 5.1. Experimental Environment and Dataset

In this experiment, we used the AutoDL cloud service platform for model training and validation. The model server’s operating system was Ubuntu 20.04, equipped with an RTX3090 GPU, 80 GB of memory, the Python 3.8 programming environment, and the PyTorch 1.13.1 deep learning framework with CUDA version 11.3. We selected the UA-DETRAC dataset as the verification dataset for the vehicle tracking analysis. The UA-DETRAC dataset was created by the Laboratory of Multimedia Computing and Intelligent Information Processing at the School of Computer Science and Technology, University of Science and Technology of China.

The UA-DETRAC dataset contains video sequences and accompanying tag files in (.xml) format. Each video sequence has a corresponding label file, detailing annotation information for all frames. The annotation includes data about multiple vehicles such as the vehicle ID, boundary frame coordinates, vehicle category, and speed.

We wrote scripts in Python to process the XML files of the UA-DETRAC dataset, aiming to extract information about the detection target and reorganize and store it in the MOT16 dataset file format. This conversion allows us to generate the required detection and ground truth files.

### 5.2. Evaluation Index

Multi-target tracking is a complex task that requires the use of a variety of evaluation indexes to assess its performance. Below are some typical multi-target tracking evaluation indicators and their calculation equations:

(1) Multiple Object Tracking Accuracy (MOTA)

MOTA considers various factors that influence tracking performance. These factors include false positives (FP), missed positives, and ID switching. It evaluates the overall performance of the tracking algorithm using a comprehensive index, and the calculation method is presented in Equation (18).

(18) $$MOTA=1-\frac{\sum \left(FN+FP+IDSW\right)}{\sum GT}$$

where *FN* represents the number of undetected real targets, *FP* represents the number of incorrectly assigned detections, *IDSW* (ID Switches) represents the number of trajectory ID changes, and *GT* represents the total number of real targets.

(2) Multiple Object Tracking Precision (MOTP)

MOTP measures the average positioning accuracy of the tracking algorithm for correctly matched objects, reflecting the accuracy of the tracker’s estimation of the target position. The calculation method is shown in Equation (19).

(19) $$MOTP=\frac{\sum_{t,i}d_{t,i}}{\sum_{t}c_{t}}$$

where *d_t,i_* represent the average metric distance between the detected target and the real box assigned to it in all frames, and *ct* represents the total number of successfully matched targets in the current frame image.

(3) IDF1 (ID F1 Score)

IDF1 is a commonly used metric for evaluating ID Fidelity in tracking tasks. It focuses on the consistency of the target’s identity during tracking. IDF1 measures recognition accuracy by calculating the maximum match between the predicted tracking results and the real data, as shown in Equation (20).

(20) $$IDF1=\frac{2IDTP}{2IDTP+IDFP+IDFN}$$

In the equation, *IDTP* (ID True Positives) represents the number of correct matches between tracking targets and detection targets, while *IDFN* (ID False Negatives) signifies the number of missed detection targets during tracking. *IDFP* (False Positives) indicates the number of false matches between tracking targets and detection targets.

### 5.3. Analysis of Experimental Results

To assess the effectiveness of the enhanced vehicle tracking algorithm described in this paper, we conducted an ablation experiment to compare various modules, as well as a comparison experiment to evaluate different algorithms. The tests were performed using a vehicle tracking dataset.

(1) Ablation experiment

To evaluate the impact of each enhanced module on vehicle tracking, we conducted ablation experiments using the UA-DETRAC dataset. We aimed to assess the effectiveness of the following improvements: the new YOLOv8 vehicle detection model (module A), the retraining of appearance features (module B), and the enhanced IOU measurement (module C). The results of the ablation experiments for each module on the UA-DETRAC dataset are presented in Table 3. In the experiments, the original model used DeepSORT as the base model ① and the YOLOv8 model as the detector. Subsequent experiments ②, ③, ④, and ⑤ involved replacing the original YOLOv8 model with the improved version and adding each module one by one to assess its impact.

The ablation experiment results in Table 3 demonstrate that the proposed modules in this paper all enhance the stability of vehicle tracking. In experiment ②, using the improved YOLOv8 as the detector led to improvements in MOTA, MOTP, and IDF1 along with a 21-fold reduction in IDSW. Experiment ③ involved loading appearance features onto the re-identification dataset after retraining, resulting in various improvements in the above three evaluation indicators and a 34-fold reduction in ID switching. This indicates that the deep appearance feature network significantly enhances the vehicle feature extraction ability after retraining, enabling accurate tracking even in the event of occlusion. In experiment ④, an improved IOU distance measurement, based on experiment ②, led to enhancements in the MOTA and IDF1. Combining the three improved modules in comparison experiment ① and experiment ⑤ increased the MOTA and MOTP by 4.6% and 3.6%, respectively, based on the original model and greatly reduced the number of ID switches by 70 times. The ablation experiments show that each improved module in this paper can enhance the DeepSORT tracker.

(2) Comparative experiments of different algorithms

To validate the enhanced algorithm proposed in this paper, we compared it with several mainstream target tracking algorithms using video sequences with heavy traffic from the converted UA-DETRAC dataset. The experimental results can be found in Table 4.

In Table 4, the test results of various tracking methods indicate that substituting the original DeepSORT model’s detector with the upgraded YOLOv8 model led to significant enhancements in all tracking metrics. Specifically, MOTA and MOTP saw respective increases of 6.2% and 4.2%, while ID switches were notably reduced. This demonstrates that a superior detection model can greatly enhance tracking performance. Furthermore, compared to mainstream target tracking algorithms such as Bytetrack [28] and StrongSORT [29], the proposed improved model in this paper exhibits advantages in MOTA, MOTP, and reduced ID switches. The experimental results on a vehicle tracking dataset illustrate the improved model’s robustness in complex traffic scenarios.

(3) Visualization of tracking effect

**Figure 13:**
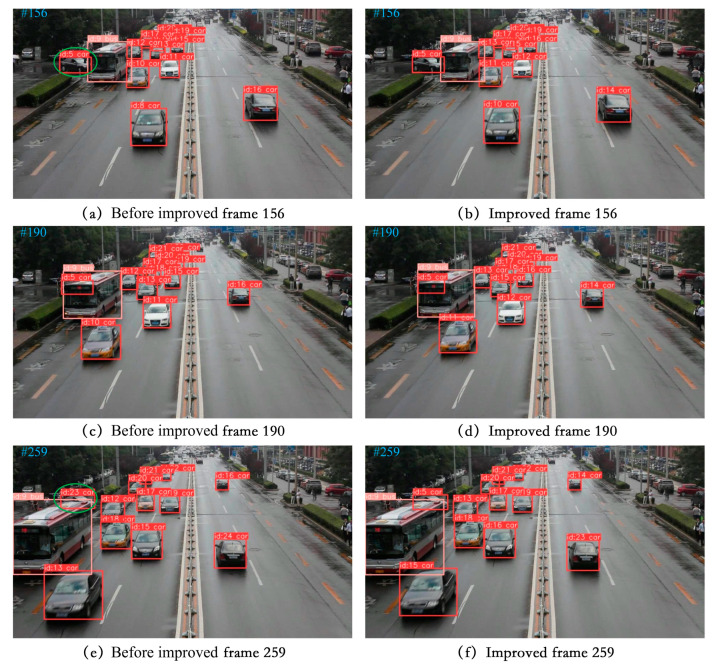
MVI_40131 video sequence tracking effect display.

**Figure 14:**
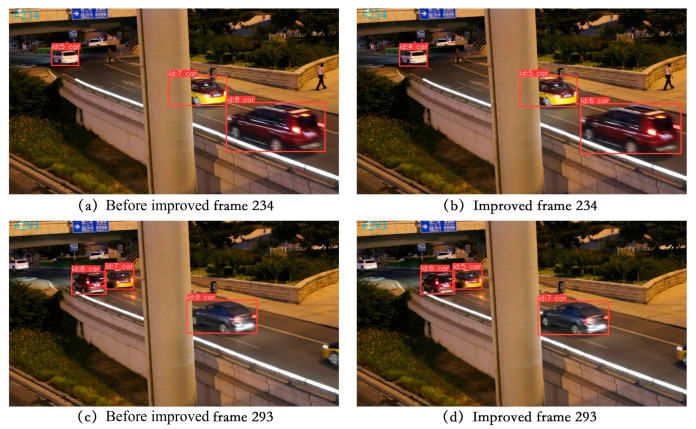
Display of MVI_40992 video sequence tracking effect.

**Figure 15:**
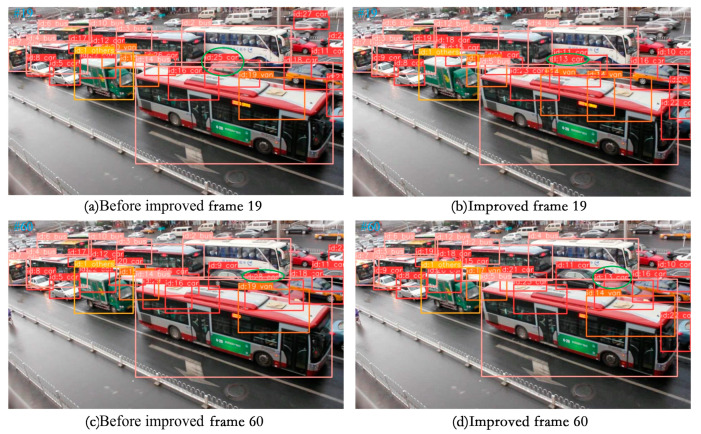
Display of MVI_40863 video sequence tracking effect.

**Figure 16:**
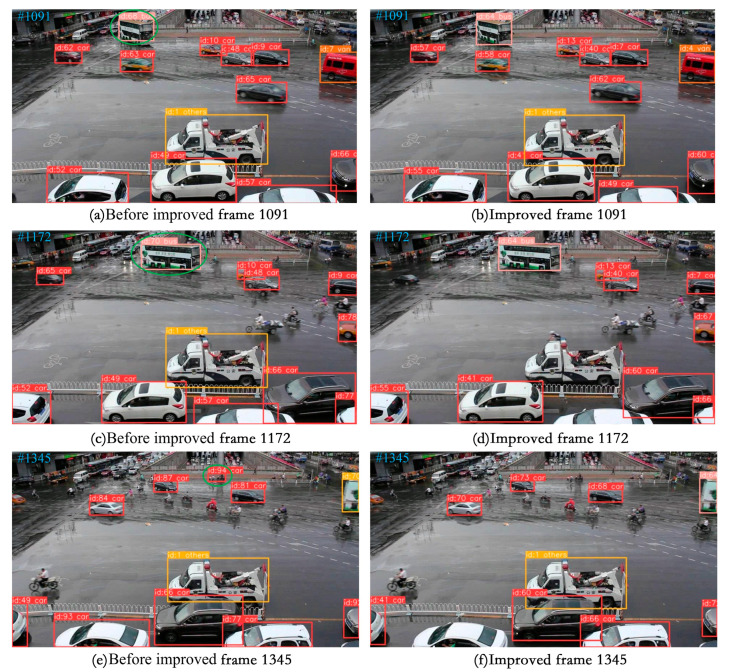
Display of MVI_40905 video sequence tracking effect.

To effectively demonstrate the tracking capabilities described in this paper, we chose a selection of video sequences from the UA-DETRAC tracking dataset as well as some self-recorded video sequences to showcase the tracking results. We specifically selected scenes with high traffic flow for testing, including night, rainy day, and occluded scenes. The four video sequences to be tested in the UA-DETRAC tracking dataset are MVI_40131, MVI_40131, MVI_40992, and MVI_40905. The tracking results are visualized in Figure 13, Figure 14, Figure 15 and Figure 16.

In Figure 13, the left and right columns represent the tracking results of the model on successive frames before and after improvement. In video sequence MVI_40131, before improvement, the vehicle with ID 5 was detected at the 156th frame. By the 190th frame, the vehicle with ID 5 was completely blocked, but its identity did not change. However, at the 259th frame, when the vehicle became partially blocked, the model reassigned a new ID to the original blocked vehicle before the improvement, causing an ID switch. On the other hand, the improved model shows a better tracking effect on the vehicle when occlusion occurs, with no ID switching.

In Figure 14, the scene of video sequence MVI_40992 is a night scene with obvious occlusion. Vehicles are partially occluded when passing by. Despite the low traffic flow in the night scene, the video sequence was separately tested on the model before and after the improvement. The tracking effect shows no ID switch occurring before and after the occlusion.

As depicted in Figure 15, the video sequence MVI_40863 was filmed on a rainy day, showing an apparent occlusion phenomenon. As illustrated, before the enhancement, the model tracked the car with ID 25 in the 19th frame, with an identity switch occurring in the target vehicle in the 60th frame. In contrast, the improved model maintains tracking stability even when the vehicle is obscured, demonstrating better performance in tracking even in scenarios such as traffic jams on rainy days.

Figure 16 shows the rainy day video sequence MVI_40905 featuring a large number of small-sized vehicles with a complex overall background. The tracking performance before and after improvement is compared. Before the improvement, the model detected a bus with ID 68 in frame 1091. However, in frame 1172, the vehicle with ID 68 changed its identity without being obscured, indicating insufficient tracker stability. Additionally, before the improvement, the model mistakenly identified bicyclists and pedestrians as vehicles with id 94 until frame 1345. Conversely, after the improvement, the model showed relatively stable tracking without identity-switching issues and performed well in complex traffic scenarios.

## 6. Conclusions

This paper begins by providing a detailed introduction to the multi-target tracking algorithm DeepSORT, covering its basic principles, the use of Kalman filter technology for target state estimation, the process of data association, and the cascade matching algorithm. Subsequently, this paper builds upon the DeepSORT algorithm to create an improved DeepSORT vehicle tracking algorithm, focusing on three key areas: optimizing the vehicle detector, refining the process for extracting appearance features, and enhancing the IOU distance measurement method. The performance of the improved algorithm was validated using a tracking dataset, and both ablation and comparison experiments were conducted to assess its effectiveness. The results demonstrate that the improved tracking algorithm exhibits more stable tracking performance and effectively reduces the occurrence of identity switching. Additionally, selected video sequences and self-compiled datasets were utilized to visually showcase the stability of the improved algorithm in tracking objects in the presence of occlusion.

## Figures and Tables

**Table 1:** Network structure of target feature extraction.

Module Type	Convolution Kernel Size/Step Size	Feature Map Output
Convolution layer 1	3 × 3/1	32 × 128 × 64
Convolution layer 2	3 × 3/1	32 × 128 × 64
Maximum pooling layer 3	3 × 3/2	32 × 64 × 32
Residual block 4	3 × 3/1	32 × 64 × 32
Residual block 5	3 × 3/1	32 × 64 × 32
Residual block 6	3 × 3/2	64 × 32 × 16
Residual block 7	3 × 3/1	64 × 32 × 16
Residual block 8	3 × 3/2	128 × 16 × 8
Residual block 9	3 × 3/1	128 × 16 × 8
Fully connected layer 10		128
L2 normalization		128

**Table 2:** Experimental environment configuration.

Category	Version Number
Operating system	Windows10
CPU	AMD Ryzen 9 5950X
GPU	NVIDIA GeForce RTX3090TI
Deep learning framework	PyTorch 1.10

**Table 3:** Results of ablation experiment.

Group	A	B	C	MOTA (%)	MOTP (%)	IDF1 (%)	IDSW
①				55.6	70.2	71.4	476
②	√			57.6	72.6	73.2	455
③	√	√		58.8	73.2	73.8	421
④	√		√	58.6	71.5	75.4	414
⑤	√	√	√	60.2	73.8	77.3	406

**Table 4:** Comparison of experimental results of different tracking methods.

Tracking Algorithm	MOTA (%)	MOTP (%)	IDF1 (%)	IDSW
SORT	46.3	67.5	62.6	1162
DeepSORT	51.4	68.4	70.2	514
YOLOv8 + DeepSORT	55.6	70.2	71.4	476
Improved YOLOv8 + DeepSORT	57.6	72.6	73.2	455
Bytetrack	57.4	72.8	75.1	452
StrongSORT	58.8	73.6	78.6	434
Ours	60.2	73.8	77.3	406

## Data Availability

All codes, data, and materials included in this research are available upon request from the corresponding author.
